# *TMTC4* is a hair cell–specific human deafness gene

**DOI:** 10.1172/jci.insight.172665

**Published:** 2023-12-22

**Authors:** Jiang Li, Byung Yoon Choi, Yasmin Eltawil, Noura Ismail Mohamad, Yesai Park, Ian R. Matthews, Jin Hee Han, Bong Jik Kim, Elliott H. Sherr, Dylan K. Chan

**Affiliations:** 1 Department of Neurology and; 2 Department of Pediatrics, Institute of Human Genetics, Weill Institute for Neurosciences, UCSF, San Francisco, California, USA.; 3 Department of Otorhinolaryngology, Seoul National University Bundang Hospital, Seoul, South Korea.; 4 Department of Otolaryngology-Head and Neck Surgery, San Francisco, California, USA.; 5 Department of Otorhinolaryngology, Chungnam National University College of Medicine, Chungnam National University Sejong Hospital, Sejong City, South Korea.

**Keywords:** Otology, Cell stress, Genetic diseases, Mouse models

## Abstract

Transmembrane and tetratricopeptide repeat 4 (*Tmtc4*) is a deafness gene in mice. *Tmtc4*-KO mice have rapidly progressive postnatal hearing loss due to overactivation of the unfolded protein response (UPR); however, the cellular basis and human relevance of *Tmtc4*-associated hearing loss in the cochlea was not heretofore appreciated. We created a hair cell–specific conditional KO mouse that phenocopies the constitutive KO with postnatal onset deafness, demonstrating that *Tmtc4* is a hair cell–specific deafness gene. Furthermore, we identified a human family in which *Tmtc4* variants segregate with adult-onset progressive hearing loss. Lymphoblastoid cells derived from multiple affected and unaffected family members, as well as human embryonic kidney cells engineered to harbor each of the variants, demonstrated that the human *Tmtc4* variants confer hypersensitivity of the UPR toward apoptosis. These findings provide evidence that *TMTC4* is a deafness gene in humans and further implicate the UPR in progressive hearing loss.

## Graphical abstract

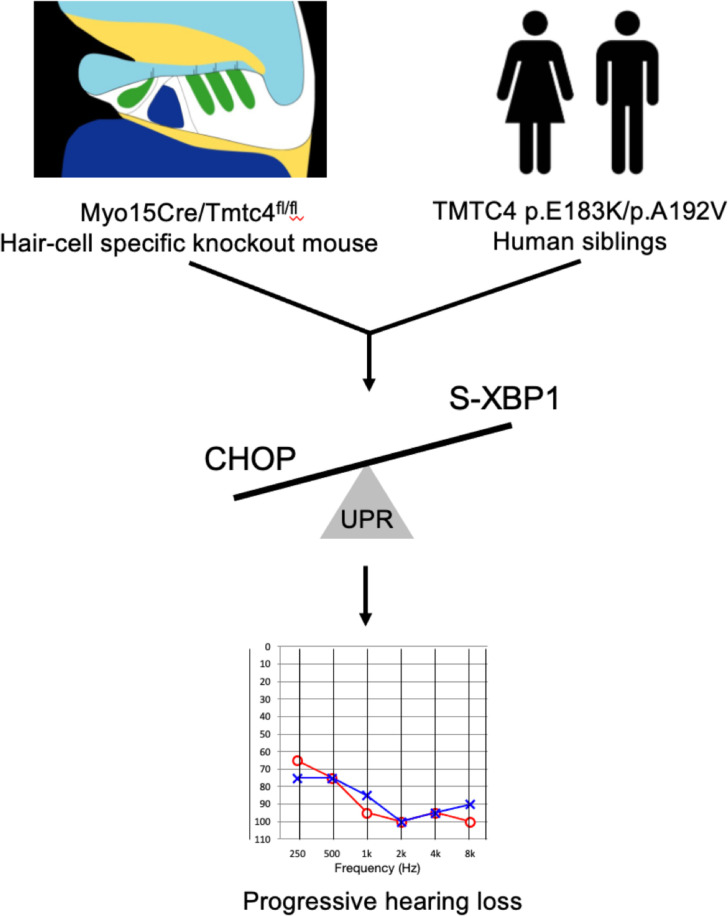

## Introduction

We recently demonstrated that transmembrane and tetratricopeptide repeat 4 (*Tmtc4*) is a recently described deafness gene in mice (1). Mice lacking Tmtc4 have normal postnatal onset of hearing, measured by auditory brainstem response (ABR) thresholds, at P13, with rapid progression to complete deafness by P26, accompanied by subsequent loss of sensory hair cells in the cochlea. Tmtc4 deficiency is associated with dysregulation of Ca^2+^ flux between the cytoplasm and endoplasmic reticulum (ER), ER stress, and the unfolded protein response (UPR) (1), and it has also been implicated in impaired O-mannosylation in cell lines (2).

Multiple lines of evidence in mice have implicated the UPR in hearing loss relating to age (3) and noise (1) — both at levels resulting in permanent hearing threshold shifts and death of sensory hair cells (1, 4) as well as at levels that cause cochlear synaptopathy and “hidden hearing loss,” where moderate noise exposure leads to preserved hair cells and hearing thresholds but loss of synapses and impaired audiologic function (5). The UPR has also been described in the mechanism of ototoxicity due to cisplatin (6) and aminoglycoside treatment (7, 8). Additionally, evidence exists that some forms of genetic human deafness are related to dysfunction of the UPR, either directly —e.g., in Wolfram syndrome (9, 10) — or indirectly — e.g., in Usher syndrome (11). The most well-studied human model of UPR-associated deafness, Wolfram syndrome due to mutations in wolframin (WFS1), however, does not translate to mice, in which WFS1-KO mice have normal hearing despite exhibiting other manifestations of Wolfram syndrome, including diabetes (12). The absence of robust and parallel genetic models of UPR-associated deafness in mice and humans makes it difficult to study the mechanisms within the cochlea by which UPR dysfunction can lead to hearing loss.

The discovery and functional characterization of *Tmtc4* as a deafness gene in mice presents a potential opportunity for a robust mouse genetic model of UPR-associated hearing loss; however, previous studies did not establish the cellular origin of the hearing-loss phenotype, and evidence of a link to human deafness was absent. We previously showed by RNA in situ hybridization that *Tmtc4* expression is present in multiple cochlear cell types in adult mice (1), whereas RNA-Seq on hair cells enriched from neonatal mouse cochlea suggests selective expression of *Tmtc4* in hair cells (13, 14). Furthermore, though other Tmtc family members, including TMTC2, have been implicated in hearing loss (15, 16), there have previously not been any humans described with *TMTC4*-associated hearing loss.

In this study, we sought to develop and characterize a Tmtc4–conditional KO (Tmtc4-cKO) mouse to understand the cell type etiology of Tmtc4-associated progressive hearing loss. We also describe the first human family with evidence for a combined TMTC4 and UPR-associated hearing loss.

## Results

### cKO of Tmtc4.

**Figure 1:**
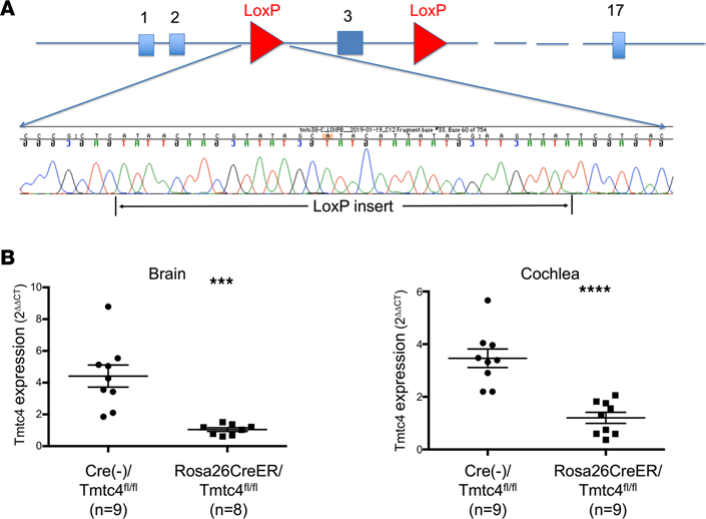
Floxed *Tmtc4* transgenic mouse (Tmtc4^fl/fl^). (**A**) LoxP sequences were inserted in the introns up- and downstream of *Tmtc4* exon 3 and confirmed by Sanger sequencing. (**B**) *Tmtc4* expression is significantly decreased in the brain and cochlea of Rosa26CreER/Tmtc4^fl/fl^ mice as compared with Cre^–^/Tmtc4^fl/fl^ mice. ***P < 0.001, ****P < 0.0001 by 1-way ANOVA.

We generated cKO mice harboring *Tmtc4* with exon 3 flanked by loxP (Figure 1). Sequencing confirmed germline transmission of the floxed allele with no off-target mutations. Carriers of 1 or 2 copies of the floxed allele breed true without morbidity when not linked to a Cre allele. For initial validation of the conditional construct, we bred floxed Tmtc4 (Tmtc4^fl/fl^) mice with mice harboring tamoxifen-inducible (TMX-inducible) ubiquitous Cre (ROSA26CreER). P3 pups were treated with TMX, and pups were sacrificed at P10. Brain and cochlea were then screened for recombination, which confirmed loss of exon 3.

**Figure 2:**
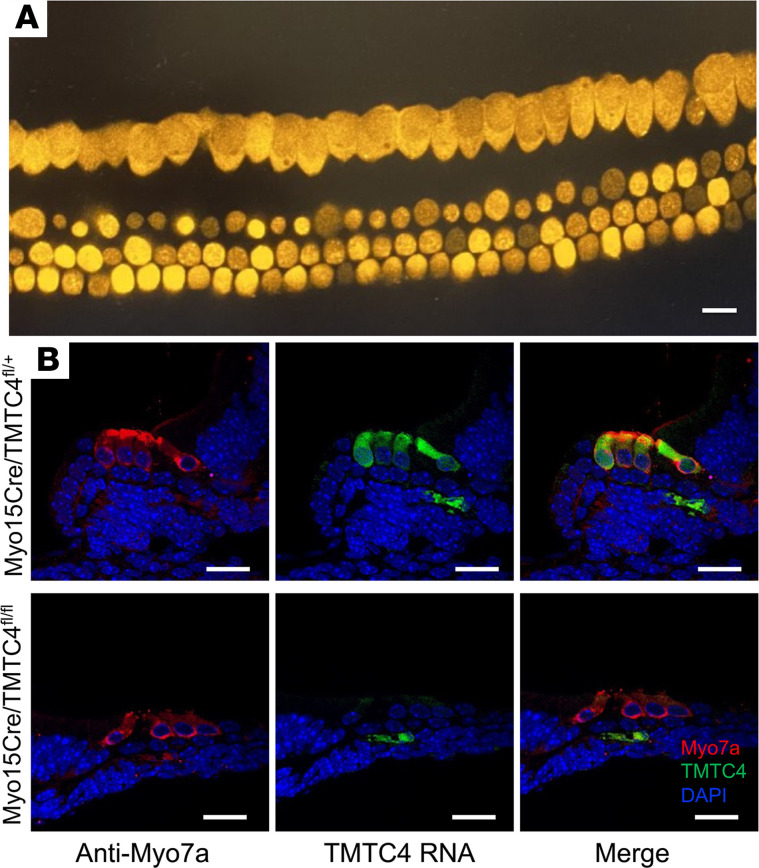
Tmtc4 expression in Myo15Cre/Tmtc4^fl/fl^ mice. (**A**) Myo15Cre/TdTomato reporter demonstrates Myo15Cre-driven TdTomato expression only in hair cells (P5 cochlear explant). (**B**) P5 cochlear explants were stained with anti-Myo7a antibody (red) to label hair cells and were subjected to RNAScope to detect Tmtc4 RNA (green). They were also stained with DAPI to identify nuclei (blue). Myo15Cre/Tmtc4^fl/+^ mouse (top) exhibited Tmtc4 expression in hair cells (colocalized with Myo7a) as well as outside of hair cells. In Myo15Cre/Tmtc4^fl/fl^ cKO mice (bottom), Tmtc4 expression was specifically lost in hair cells. Representative images from 3 experiments for each condition are shown.

We then established a Myo15Cre/Tmtc4^fl/fl^ line and validated *Tmtc4* knockdown using RNAScope, with *Myo7a* as a hair cell marker and *Chop* as a concurrent marker of proapoptotic UPR activation (Figure 2). Antibody against mouse Tmtc4 is not available; therefore, we validated hair cell–specific recombination and TMTC4 knockdown using the Myo15Cre line in 2 ways. First, we generated reporter mice that express TdTomato only in cells expressing Cre recombinase (Myo15Cre/TdTomato). Cochlear explants from these mice demonstrated TdTomato expression only in hair cells (Figure 2A). Second, we performed RNAScope in situ hybridization against Tmtc4, with simultaneous IHC using anti-Myo7a antibody as a marker for hair cells. This demonstrated that, in Myo15Cre/Tmtc4^fl/+^ mice, there was extensive colocalization of Tmtc4 puncta with anti-Myo7a antibody in hair cells as well as some Tmtc4 expression outside of hair cells. In the homozygous cKO (Myo15Cre/Tmtc4^fl/fl^), TMTC4 puncta were absent in Myo7a^+^ cells but still present in supporting cells (Figure 2B). This demonstrates that Myo15Cre/Tmtc4^fl/fl^ mice had hair cell–specific KO of Tmtc4.

### Hearing loss in TMTC4-cKO mice.

**Figure 3:**
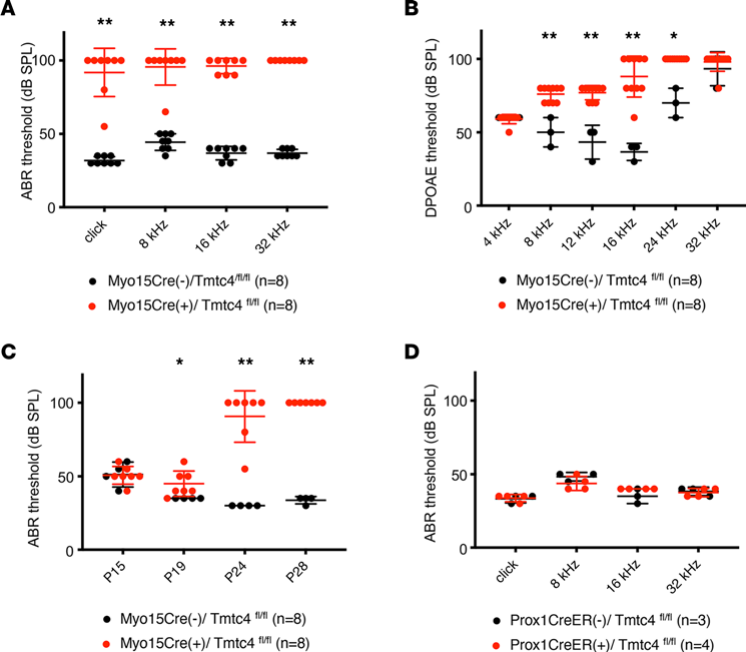
Auditory function in Tmtc4-cKO mice. (**A** and **B**) ABR (**A**) and DPOAE (**B**) thresholds are elevated in adult (>P30) cKO mice in which Tmtc4 is subject to recombination by Cre driven by the hair cell–specific Myo15Cre promoter (Myo15Cre^+^/Tmtc4^fl/fl^), compared with Cre^–^ littermate controls (Myo15Cre^–^/Tmtc4^fl/fl^). (**C**) Progression of hearing loss is seen in Myo15Cre/Tmtc4^fl/fl^ cKO mice from onset of hearing (P15) through P28. (**D**) ABR thresholds in adult (>P30) Prox1CreER^+^/Tmtc4^fl/fl^ mice, in which Tmtc4 is specifically knocked out in supporting cells, are not elevated compared with Cre^–^ controls (Prox1CreER^–^/Tmtc4^fl/fl^). *P < 0.05; **P < 0.001, 2-tailed unpaired Student’s *t* test between genotypes.

We measured ABR and distortion product otoacoustic emissions (DPOAE) in Myo15Cre/Tmtc4^fl/fl^ mice. Homozygous cKO mice (Myo15Cre/Tmtc4^fl/fl^) exhibited normal ABRs and DPOAEs at auditory onset (P13) and rapid progression to complete deafness by P26, similar to constitutive Tmtc4-KO mice (1). In contrast, Cre^–^ littermates had normal hearing into adulthood (Figure 3, A–C, and Supplemental Figure 1A; supplemental material available online with this article; https://doi.org/10.1172/jci.insight.172665DS1). cKO of Tmtc4 using other Cre drivers corroborated the conclusion that Tmtc4-KO–associated hearing loss is a hair cell–specific phenotype; Atoh1Cre, which induces recombination in hair cells as well as supporting cells, also gave rise to profound hearing loss (Supplemental Figure 1). In contrast, Prox1CreER, induced with TMX at P16 — which causes Cre recombination only in the supporting pillar and Deiters cells — did not affect hearing at all by P45 (Figure 3D). Cre^+^/Tmtc4^fl/+^ mice had identical hearing to Cre^–^ mice (Supplemental Figure 1).

**Figure 4:**
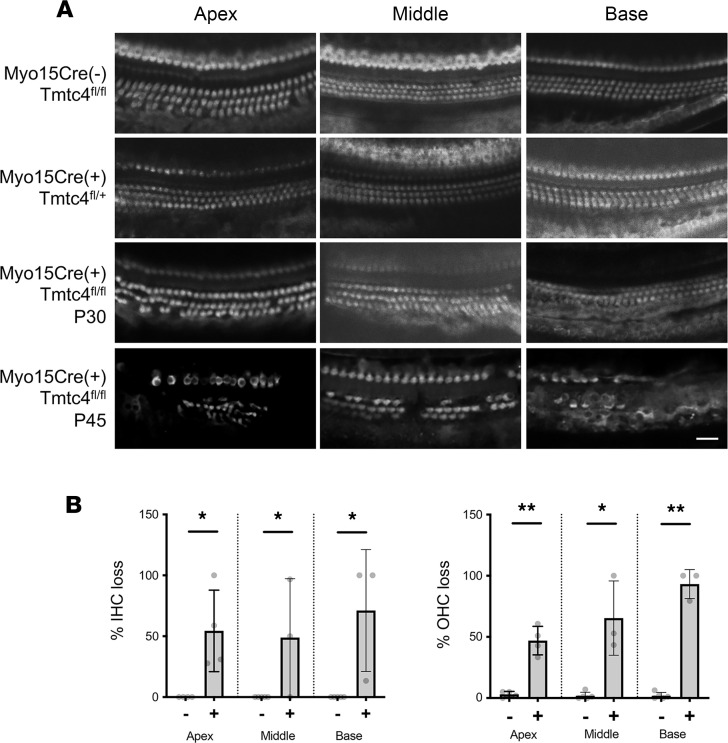
Hair cell loss in Myo15Cre/Tmtc4-cKO mice. (**A**) Whole-mount IHC against Myo7a to label hair cells was performed at the apical, middle, and basal turns of P30 and P45 Myo15Cre/Tmtc4-cKO mice of the indicated genotypes. Scale bar: 50 μm. (**B**) Hair cell loss in Myo15Cre/Tmtc4-cKO mice. Inner and outer hair cell (IHC, left; OHC, right) counts were made in the apical, middle, and basal turns of cochleae from P45 Myo15Cre^–^ (*n* = 4) and Cre^+^/Tmtc4^fl/fl^ (*n* = 4) mice. *P < 0.05; **P < 0.001 by 2-tailed unpaired Student’s *t* test between genotypes.

Quantification of inner and outer hair cells reflected the auditory findings seen in Myo15Cre/Tmtc4^fl/fl^-cKO mice. Whereas Myo15Cre^–^/Tmtc4^fl/fl^ and Myo15Cre/Tmtc4^fl/+^ littermates showed no loss of hair cells through P45, Myo15Cre/Tmtc4^fl/fl^ homozygous cKO mice had intact hair cells through P30 but partial hair cell loss in all 3 cochlear turns at P45 (Figure 4 and Supplemental Figure 2).

### Human hearing loss is associated with autosomal recessive TMTC4 variants.

**Figure 5:**
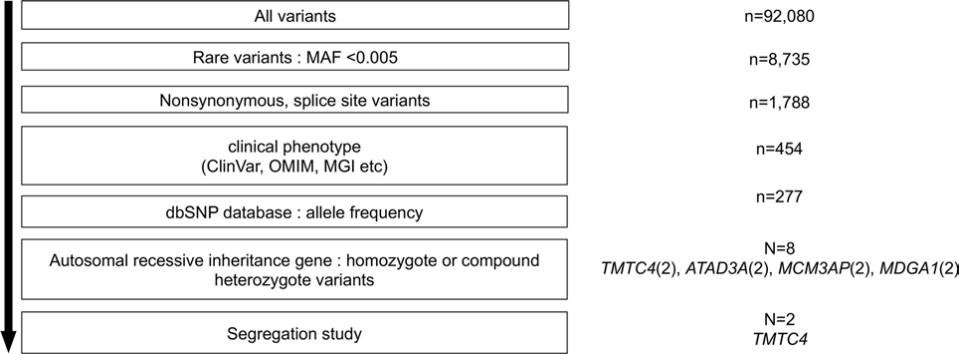
Identification of human *TMTC4* variants. Variants from exome sequencing data (*n* = 92,080) underwent filtering steps. After each filtering step, candidate variants were narrowed down to 8 candidates: 2 variants in 4 genes (*TMTC4*, *ATAD3A*, *MCM3AP*, *MDGA1*). After Sanger sequencing confirmation and segregation study, 2 variants of *TMTC4* remained as potential causative variants.

Two affected individuals in a Korean family — each with history of progressive sensorineural hearing loss (SNHL) over their lives, albeit to different degrees — were found on whole-exome sequencing to be compound heterozygous for 2 candidate pathogenic missense variants in *TMTC4* (c.547 G>A: p.Glu183Lys [p.E183K; Combined Annotation Dependent Depletion (CADD) phred 27.00] and c.575 C>T: p.Ala192Val [p.A192V; CADD phred 31.00]), while the parents and the unaffected sibling each only carried one of these variants (Figure 5). Detailed information including the in silico and population data of these 2 variants is provided in Table 1.

Although the *TMTC4* gene did not undergo a formal curation process by the ClinGen Gene Curation working group (17) to be established as a deafness causing gene, we analyzed *TMTC4* using the Standard Operating Procedure of the Gene Clinical Validity Curation Process (version 9) to determine that it meets “moderate” support evidence to be a deafness-associated gene. In brief, 2 in trans missense variants scored 0.5 point each, based on functional data using a patient-derived cell line resulting in 1 point in total as genetic evidence. Mouse models, including our published Tmtc4-KO (1), and cell lines engineered to harbor each of the variants (see below) collectively provide 6 points, the highest score in terms of experimental evidence. Combining genetic and experimental evidence, the strength of the accumulated evidence would reach a “moderate support” level to establish a gene-disease relationship — specifically, a relationship between TMTC4 and hearing loss. Assuming this relationship, using American College of Medical Genetics and Genomics (ACMG) and Association for Molecular Pathology (AMP) guidelines (18, 19) and Rare Exome Variant Ensemble Learner (REVEL) and CADD scores, these variants would be classified as “likely pathogenic” (Table 1).

**Figure 6:**
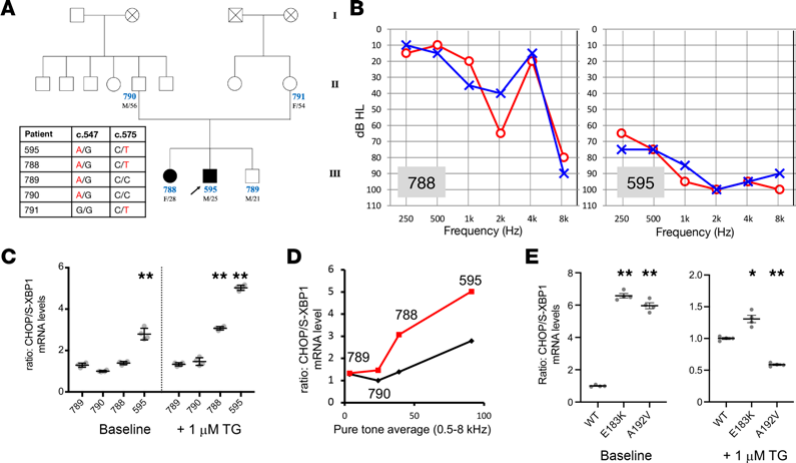
TMTC4 variants in human hearing loss. TMTC4 is the likely cause of nonsyndromic progressive sensorineural hearing loss. (**A**) Three-generation pedigree consistent with autosomal recessive inheritance pattern. Black: hearing loss; white: normal hearing; cross: deceased. Genotyping at c.547 and c.575 loci of TMTC4 demonstrates cosegregation of compound heterozygous rare variants c.547 G>A and c.575 C>T with hearing loss in patients 788 and 595 (pathogenic variants in red). (**B**) Audiograms for patients 788 and 595 show noise-induced notch at 2 kHz and high-frequency–predominant sensorineural hearing loss. (**C**) Lymphoblastoid cell lines were established from 4 human family members. mRNA levels of CHOP and S-XBP1 (opposing proapoptotic and prohomeostatic effectors of the UPR, respectively) were measured by qPCR, and the CHOP/S-XBP1 ratio, which reflects the proapoptotic balance of the UPR, was calculated. *n* = 4 for each condition. One-way ANOVA followed by Tukey’s multiple-comparison test was performed, with pairwise comparisons relative to patient 789 (as a normal-hearing control) performed. At baseline (left), only patient 595 had significant elevation of the CHOP/S-XBP1 ratio. After treatment with 1 mM thapsigargin (TG) for 6 hours, cells from the 2 patients with hearing loss (788 and 595) had significant elevation of the CHOP/S-XBP1 ratio. **P < 0.001. (**D**) CHOP/S-XBP1 ratio for the 4 patients was correlated with hearing level (bilateral pure-tone average to 0.5–8 kHz tones). The correlation was not significant for baseline CHOP/S-XBP1 ratio (black line) but was statistically significant for thapsigargin-induced levels (red line; linear regression *R*^2^ = 0.94, *P* < 0.05). (**E**) HEK cells were generated harboring homozygous E183K or homozygous A192V mutations, and CHOP and S-XBP1 mRNA levels were measured as in **C**. E183K and A192K mutant cells exhibited elevated CHOP/S-XBP1 ratios at baseline (left). Upon UPR induction with 1 μm TG (right), E183K cells had a greater CHOP/S-XBP1 ratio, whereas A192V cells had a lower CHOP/S-XBP1 ratio, compared with WT cells. *n* = 4 for each condition. *P < 0.01; **P < 0.001 by 2-tailed unpaired Student’s *t* test relative to WT.

Lymphoblastoid cells were derived from 2 affected siblings, the unaffected sibling, and 1 unaffected parent (Figure 6, A and B). We tested the levels of CHOP and S-XBP1 mRNA in these cells, as markers of the proapoptotic and prohomeostatic arms of the UPR, respectively. Quantitative PCR (qPCR) analysis revealed that cells from the affected siblings had a significantly increased ratio of CHOP to S-XBP1 expression, reflecting balance toward apoptosis, both at baseline and upon UPR induction with thapsigargin (*P* < 0.001). The thapsigargin-induced increase in CHOP/S-XBP1 ratio correlated strongly with pure-tone average in the 4 patients (*P* < 0.05, *R*^2^ = 0.94) (Figure 6, C and D).

To further confirm the functional effect of the human variants, a homozygous p.E183K and p.A192V variant, respectively, of *TMTC4* was introduced into HEK cells using CRISPR/Cas9-mediated recombination. Similar to the cells from patients with hearing loss and *TMTC4* missense variants, TMTC4 p.E183K and p.A192V HEK cells were found to have an elevated ratio of CHOP/S-XBP1 mRNA at baseline (*P* < 0.001). Upon UPR induction with 1 μM thapsigargin, p.E183K cells showed an increase in the CHOP/S-XBP1 mRNA ratio, whereas p.A192V cells showed a decrease (*P* < 0.01 and *P* < 0.001, respectively; Figure 6E).

## Discussion

*Tmtc4* is a progressive hearing-loss gene. In mice, Tmtc4 deficiency permits normal initial cochlear development and hearing maturation at P13, but it leads to rapid progressive hearing loss due to overactivation of the apoptosis arm of the UPR (1). Here, we demonstrate successful development of cell type–specific cKO of Tmtc4. Using these mice, we show that Tmtc4-associated hearing loss is a hair cell–specific phenotype. This demonstrates the importance of Tmtc4 and the UPR in hair cell physiology and establishes that the hearing loss caused by Tmtc4 deficiency originates from the sensory hair cells, rather than being a secondary sequela of dysfunction of another cell type within the cochlea. This genetic mouse model will be useful for future studies of progressive hearing loss due to UPR dysfunction and to investigate subsequent rescue by targeting the UPR in hair cells (1, 5).

As in the constitutive KO (1), hearing in Myo15Cre-TMTC4–cKO mice developed normally, hearing loss was noted almost immediately after the onset of hearing, and mice were completely deaf by P26. In contrast to the constitutive KO, both inner and outer hair cells were largely preserved at P30 and were only partially lost by P45. This suggests that Tmtc4 does not play a direct functional role in hair cell development or function and is, instead, consistent with a critical role in hair cell maintenance or repair, through its involvement in the UPR (1) and glycosylation (2). One possible mechanism that may explain how hearing is normal at the onset of hearing but rapidly declines may be related to tip-link stability and regeneration. Cadherins are targets of TMTC family member O-mannosylation, and Cdh23 is 1 of the 2 principal components of the tip link that connects adjacent stereocilia in the apical hair bundle to gate mechanotransduction channels. Upon tip-link disruption, Cdh23 — unlike its tip-link partner, PCDH15 — is not retained in the stereocilia (20) but is instead likely trafficked to the ER for refolding and/or glycosylation. Aberrant glycosylation of Cdh23 may cause tip-link destabilization in Tmtc4-deficient hair cells, leading to rapid tip-link disruption and a functional deficit causing hearing loss without initial hair cell death. The absence of Tmtc4 could further impair refolding and glycosylation of disrupted tip links, and the associated hypersensitization of the UPR could then secondarily induce hair cell death. The detailed mechanism by which Tmtc4 may lead to dysfunction and, ultimately, death of hair cells should be investigated in future studies.

In this study, we also provide, to our knowledge, the first genetic and functional evidence that *TMTC4* is involved in human hearing, by describing 2 missense variants of *TMTC4* in humans that segregate with progressive hearing loss in a single family. Interestingly, among the affected members, the younger brother (SB296-595) showed worse hearing than his older sister (SB296-788), and cells from his blood showed a higher CHOP/S-XBP1 ratio. While the association between a higher CHOP/S-XBP1 ratio and more impaired hearing may be causally related, there are other potential reasons for worse hearing loss in patient SB296-595, since he is male and had greater noise-exposure history, working for a large amusement park as a field worker. It is possible that this noise exposure history may have contributed to his hearing deterioration. We have implicated the UPR in noise-induced hearing loss (1); in this individual, therefore, the effects of noise exposure and UPR hypersensitivity conferred by *TMTC4* mutations may have had a synergistic effect on his hearing loss.

Functional analysis of cells from affected probands and unaffected family members, as well as HEK cells engineered to harbor each of the missense variants in homozygous state, show that these cells are hypersensitive to UPR upregulation toward the proapoptotic CHOP arm, consistent with the hypothesis that these *TMTC4* missense variants affect the UPR. Though each variant affected thapsigargin-induced UPR activation in different ways compared with WT cells, these results clearly demonstrate that these TMTC4 variants modulate the UPR. These results together provide moderate evidence that *TMTC4* is a deafness gene in humans, causing progressive hearing loss due to UPR dysregulation in humans. To more definitively establish this gene-disease relationship, additional families with hearing loss harboring *TMTC4* variants would need to be identified, with formal curation through ClinGen.

### Conclusion.

In this study, we demonstrate that Tmtc4 deficiency specifically in hair cells causes progressive postnatal hearing loss in mice, a phenotype that is also seen in humans with TMTC4 missense variants. These findings establish TMTC4 deficiency as a clear example of genetic UPR-associated hearing loss that can be used as a model for how the UPR affects hearing and deafness in mice and humans.

## Methods

### Genetic models.

We used CRISPR/Cas9 to introduce loxP sequences flanking exon 3 of *Tmtc4*. A construct containing CRISPR/Cas9 and a ~700 bp ssODN template was injected into mouse zygote to result in a homology directed repair, resulting in exon 3 being flanked by loxP sequences. The guide DNA sequence upstream of exon 3 was 5′-ACAGTGAGCCGTGAGGAGAGCGG-3′ and downstream was 5′-CAAGATGCCAGCACACTCTGAGG-3′. Final creation of the floxed Tmtc4 mouse (Tmtc4^fl/fl^) was performed by the UC Davis Mouse Biology Program (Davis, California, USA). *Tmtc4* expression was tested in brain and cochlear tissue isolated from Tmtc4^fl/fl^ mice bred with Rosa26CreER (B6.Cg-*Gt[ROSA]26Sor^tm1.1[CAG-rtTA3]Slowe^*/LdownJ; The Jackson Laboratory, no. 029627) mice to induce ubiquitous Cre-mediated recombination. Total RNA was isolated using TRIzol (Thermo Fisher Scientific, 15596026), and 1 μg total RNA was used for first-strand cDNA synthesis using the SuperScript IV VILO master mix (Thermo Fisher Scientific, 11756050). Real-time PCR was performed by Bio-Rad CFX384 qPCR system, with Taqman assay for Tmtc4 (Mm01226759_m1; Thermo Fisher Scientific).

To introduce a homozygous p.E183K and p.A192K variant, respectively, into human *TMTC4*, engineered cell clones were created using chemically modified synthetic sgRNA and SpCas9 transfected as RNPs to ensure high editing efficiencies. The ssDNA of the donor sequences were as follows: E183K: 5′-CTCGCCCCCAGGGCGTCCCTGCTGGCCGCGCTGCTGTTTGCTGTCCATCCTGTCCACACCAAGTGTGTAAGTGTCGTCTGCCCGAGGCATCTGTATGCACGTGAGAAGCGATATGACACCC-3′; and A192V: 5′-ATTCTTTGTTTTCTTCCCCCTTTTTTAAGGTTGCTGGTGTTGTCGGCCGTGTAGACCTCCTGTGTGCCCTGTTCTTCTTGTTATCTTTCCTTGGCTACTGT-3′. Final creation of the cell line was conducted by Synthego.

TdTomato reporter mice were purchased from The Jackson Laboratory (*Gt[ROSA]26Sor^tm14[CAG-tdTomato]Hze^*, no.007914). When bred to Myo15Cre mice, the resulting offspring have the STOP cassette deleted in the *Cre*-expressing cochlea, resulting in robust tdTomato fluorescence. *Tmtc4^fl/fl^* mice were bred with ROSA26CreER (B6.Cg-*Gt[ROSA]26Sor^tm1.1[CAG-rtTA3]Slowe^*/LdownJ; The Jackson Laboratory, no. 029627), Atoh1Cre (B6.Cg.Tg[Atoh1-cre]1Bfri/J; The Jackson Laboratory, no. 011104), Myo15Cre (21) (gift from S. Heller, Stanford University, Palo Alto, California, USA), and Prox1CreER (*Prox1^tm3[cre/ERT2]Gco^*/J; The Jackson Laboratory, no. 022075) for inducible/ubiquitous, hair cell/supporting-cell nonspecific, hair cell–specific, and inducible/supporting cell–specific Tmtc4 knockdown, respectively.

### Human genetics.

One proband (M/25) presented with bilateral profound SNHL, while his older sister (F/28) had bilateral moderate midfrequency SNHL. Exome sequencing was performed from blood from 1 proband (M/25), and subsequent bioinformatics analysis was done to narrow down the candidate variants as we have described previously (22). In brief, called variants were filtered out with the cutoff of MAF < 0.005, and nonsynonymous or splice site variants were chosen. Next, variants with matching clinical phenotypes using ClinVar, OMIM, or MGI were filtered in, and allele frequency based on dbSNP and inheritance pattern were considered (Figure 5).

Potential candidate variants were Sanger sequenced and segregated with hearing loss in affected and unaffected family members to determine the causative variants. Lymphoblastoid cells were derived from these individuals. Thapsigargin (1 μM) was applied to cells for 6 hours to induce ER calcium depletion and UPR activation. Expression levels of 3 UPR markers (CHOP, correlated with proapoptotic activity of the UPR; S-XBP1, also associated with prohomeostatic activity of the UPR) were measured by qPCR and quantified against GAPDH and unexposed controls using the 2^−ΔΔCT^ method. The ratio of CHOP/S-XBP1 was used as a marker of the proapoptotic state of the UPR. One-way ANOVA followed by Tukey’s multiple-comparison test was performed, with pairwise comparisons for each proband relative to their normal-hearing relatives. Linear regression was used to measure the association between CHOP/S-XBP1 ratio and pure-tone average in tested family members.

### Auditory testing.

Hearing was tested in mice by measuring ABR thresholds in response to broadband tone pips at 8, 16, and 32 kHz in the sound field and by measuring DPOAE from 4–32 kHz in closed field, using a standard commercial system (RZ6, Tucker-Davis Technologies) in a soundproof chamber as described (1). Hearing was tested from the onset of hearing at P13 through P45. All auditory measurements were performed by an investigator masked to genotype.

### IHC.

Evaluation of hair cell loss was performed with whole-mount cochlear IHC as described (1). Cochleae from *Tmtc4*-cKO mice and littermates at varying time points were isolated, fixed, and decalcified. Following decalcification, the otic capsule was then dissected and incubated with anti-Myo7a antibody (a hair cell–specific marker; 1:50 dilution in PBS; Proteus Biosciences, 25-6790) and incubated overnight at 4°C. Whole-mount cochleae were rinsed twice for 10 minutes with PBS and then incubated for 2 hours with a goat anti–rabbit IgG antibody conjugated to Cy2 (1:2,000 dilution in PBS; Jackson ImmunoResearch, 111-165-003). Whole mounts were rinsed in PBS twice for 15 minutes, further microdissected into individual turns for surface preparation, and mounted with VectaShield (Vector Laboratories) containing DAPI to mark nuclei. Hair cells in the organ of Corti were visualized under a confocal microscope (Nikon A1R).

### In situ hybridization using RNAScope.

In order to validate Tmtc4 expression in WT and KO murine models, *RNAScope* (Advanced Cell Diagnostics) was conducted on P4 cochleae. Organotypic cultures of neonatal cochleae were established as described (1). Briefly, P4 mice were euthanized and decapitated. Dissection was performed in HBSS. The cochlear duct was isolated, opened, and plated on glass coverslips with Cell-Tak (Corning, 354240) with the apical surface of the epithelium facing up. Cultures were incubated at 37°C and 5% CO_2_ in DMEM-F12 + 10% FBS and 50 mg/mL ampicillin and were used for experiments after 24 hours in culture.

*RNAScope* probe Mm-Tmtc4-O1-C2 (catalog 1122531-C2), a 10ZZ probe targeting NM_028651.3:171-637, was designed to detect all validated transcript variants of mouse Tmtc4 (NR_153663.1, NM_028651.3, NM_001360598.1, and NM_001360559.1). Cochleae were isolated and fixed in 10% NBF (Neutral Buffered Formalin) overnight at 4°C. After 24 hours, cochleae were washed in PBS, and serial sucrose gradients in OCT were performed (Sakura). Cochlea were frozen using dry ice and then cryo-sectioned in a sterilized Cryo-Stat (23). The Advanced Cell Diagnostics (ACD) protocol, along with the fluorescent detection kit, was used for hybridization. In order to colabel with antibodies, after the last step of in situ hybridization, samples were washed 3 times for 5 minutes each with PBS and incubated in primary antibody in 0.1% saponin (MilliporeSigma, 47036) with PBS overnight at 4°C. After approximately 24 hours, samples were washed 3 times for 5 minutes each with PBS and incubated in secondary antibody in 0.1% saponin (MilliporeSigma, 47036) with PBS for 1 hour at room temperature. Samples were then mounted using Prolong Diamond media (Thermo Fisher Scientific, P36965). Imaging was carried out at every reiteration of the experiment with identical microscope settings (gain and laser power) using a Nikon A1R microscope. Cryosections were imaged using a 60× objective on a Nikon A1R confocal microscope. All image processing was performed in Fiji and Adobe Illustrator 2022.

### Statistics.

For comparison between treatment groups for UPR gene expression, we used 1-way ANOVA, followed by Tukey’s multiple-comparison test. For pairwise comparison of ABR thresholds between groups of mice, we used unpaired 2-tailed Student’s *t* test. Unless otherwise mentioned, results are presented as mean ± SD, with sample sizes and *P* values between designated comparison groups as indicated in the figure legends. *P* < 0.05 was considered significant, and lower *P* values are indicated for specific comparisons. Statistical analyses were performed with GraphPad Prism 7.

### Study approval.

Patient research in this study was approved by the IRB of Seoul National University Bundang Hospital (IRB-B-1007-105-402), and written informed consent was obtained from all patients. Animal research in this study was approved by the IACUC of UCSF (AN1999783-00).

### Data availability.

All underlying data and supporting analytic code used in this study will be shared upon reasonable request. Values for all data points in graphs are reported in the Supporting Data Values file.

## Author contributions

Initial design was contributed by JL, BYC, BJK, EHS, and DKC. Experimental and ethical oversight and funding were contributed by BYC, BJK, EHS, and DKC. Experimental contributions were contributed by JL, BYC, YE, NIM, YP, IRM, JHH, BJK, and DKC. Data and statistical analysis was contributed by JL, BYC, YE, NIM, BJK, DKC, and EHS. Manuscript drafting was contributed by JL, BYC, YE, BJK, DKC, and EHS. First and corresponding authors include the following: JL and BYC share first position, and BJK, EHS, and DKC share final position. This work was a collaboration between groups focused on human genetics (conducted by BYC and BJK) and mouse genetics, auditory physiology, and biochemistry (conducted by JL, EHS, and DKC). Because the animal work constituted > 50% of the actual content in the study, JL is listed first among the shared first position, and EHS and DKC are listed last among the shared final position. Between EHS and DKC, DKC is listed last, as he oversaw the entire study and was the principal investigator on the grant that supported the majority of this work.

## Supplementary Material

Supplemental data

Supporting data values

## Figures and Tables

**Table 1:**
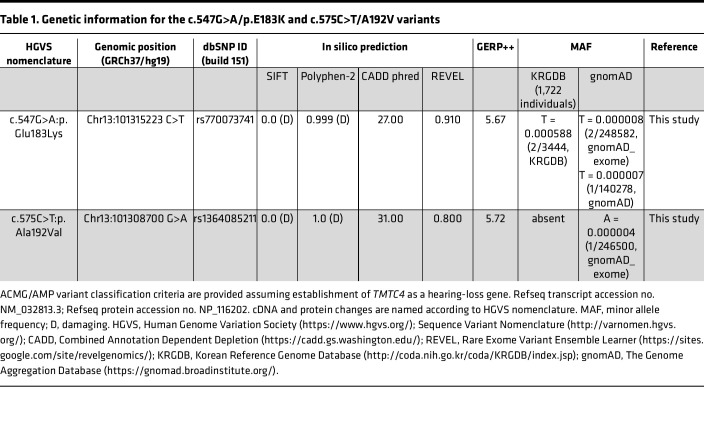
Genetic information for the c.547G>A/p.E183K and c.575C>T/A192V variants

## Version 1 11/09/2023

In-Press Preview

## Version 2 12/22/2023

Electronic publication
