# Supplementary material for: TMTC4 is a hair cell–specific human deafness gene
Source: JCI Insight. 2023 Dec 22;8(24):e172665. doi: 10.1172/jci.insight.172665 (PMC10807715; doi:10.1172/jci.insight.172665)
Supplement: Supplemental data [file jciinsight-8-172665-s056.pdf]

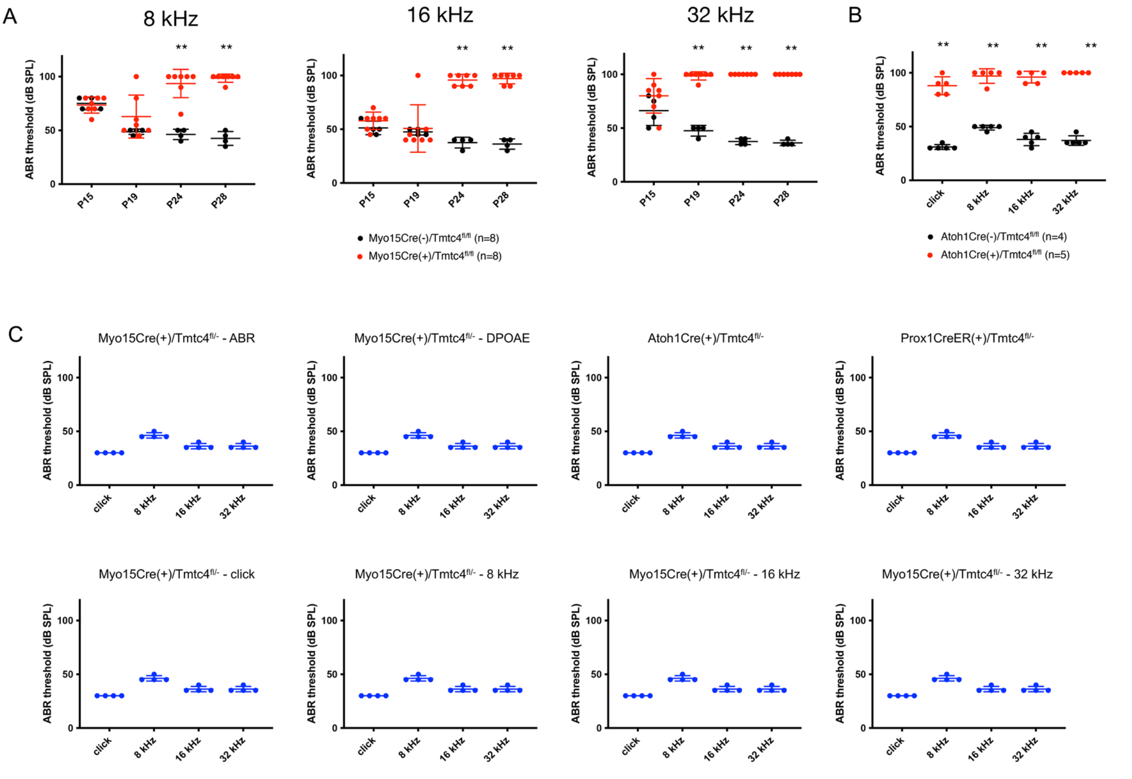

A
8 kHz
16 kHz
32 kHz
B
ABR threshold (dB SPL)
P15
P19
P24
P28
click
8 kHz
16 kHz
32 kHz
Myo15Cre(-)/Tmtc4fl/fl (n=8)
Myo15Cre(+)/Tmtc4fl/fl (n=8)
Atoh1Cre(-)/Tmtc4fl/fl (n=4)
Atoh1Cre(+)/Tmtc4fl/fl (n=5)
C
Myo15Cre(+)/Tmtc4fl/- - ABR
Myo15Cre(+)/Tmtc4fl/- - DPOAE
Atoh1Cre(+)/Tmtc4fl/-
Prox1CreER(+)/Tmtc4fl/-
Myo15Cre(+)/Tmtc4fl/- - click
Myo15Cre(+)/Tmtc4fl/- - 8 kHz
Myo15Cre(+)/Tmtc4fl/- - 16 kHz
Myo15Cre(+)/Tmtc4fl/- - 32 kHz

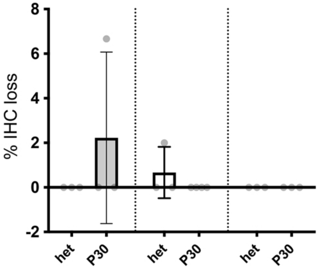

% IHC loss
8
6
4
2
0
-2
het
P30
het
P30
het
P30

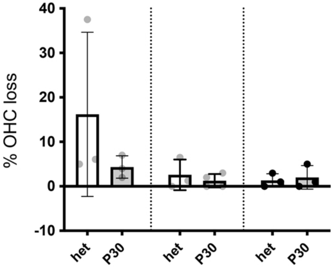

% OHC loss
40
30
20
10
0
-10
het
P30
het
P30
het
P30
